# Adult-onset perianal Langerhans cell histiocytosis presenting as pruritus ani: a case report and review of the literature

**DOI:** 10.1186/s13256-021-02924-0

**Published:** 2021-07-22

**Authors:** Marah Hamdan, Jesse C. Qiao, Vid Fikfak

**Affiliations:** 1 Departments of Surgery, Texas Tech University Health Sciences Center, El Paso, TX USA; 2 Pathology at Texas Tech University Health Sciences Center, El Paso, TX USA

**Keywords:** Langerhans cell histiocytosis, Langerhans cell sarcoma, Perianal Langerhans histiocytosis, Case report

## Abstract

**Background:**

Langerhans cells belong to the histiocytic system and give rise to two tumors: Langerhans cell histiocytosis and Langerhans cell sarcoma. Clinical aggressiveness and degree of atypia distinguish the two neoplasms. Langerhans cell histiocytosis can infiltrate a single or multiple organ systems and particularly affects bone, skin, and lymph nodes. Perianal cutaneous Langerhans cell histiocytosis is a rare condition in adults, with 15 cases reported in the literature.

**Case:**

We present the case of a 50-year-old hispanic man who presented with a 9-month history of pruritus ani and a personal history of diabetes insipidus. Punch biopsy confirmed a lesion of Langerhans cells origin but could not exclude Langerhans cell sarcoma because of limited sample size. An additional biopsy was planned as well as a positron emission tomography scan to determine the extent of disease spread. While the patient failed to follow up for repeat biopsy, the positron emission tomography scan was performed and was negative for metastatic disease. A stable perianal lesion of Langerhans cell histiocytosis with benign clinical features in a 50-year-old male despite lack of treatment is extremely rare and has not been described in the literature so far. Here, we review the presentation and workup of patients with Langerhans cell histiocytosis, review the relevant literature, and discuss treatment planning.

**Conclusion:**

Perianal Langerhans cell histiocytosis is rare, and there should be a high index of suspicion with chronic or new perianal lesions, especially in a patient with a history of diabetes insipidus. It is also important to consider the patient’s full clinical course when it is not possible to reach a definitive pathological diagnosis before management.

## Introduction

Langerhans cell histiocytosis (LCH) and Langerhans cell sarcoma (LCS) both originate from Langerhans antigen-presenting cells. LCS is clinically aggressive and extremely rare with an incidence of 0.2 per 10 million adults in the US, while LCH is less aggressive and has an incidence of 8–9 and 1–2 cases per million in children and adults, respectively [1]. LCH has a wide spectrum of clinical presentations and can be limited, affecting a single system (SS-LCH), or potentially terminal, affecting multiple systems (MS-LCH) [2]. SS-LCH is present in over 60% of patients, preferentially affecting bone (85%), skin (11%), and lymph nodes (2%) [3]. MS-LCH disseminates into the liver, spleen, and bone marrow [4]. Severe MS-LCH has been associated with BRAF V600 mutation and has been treated with mercaptopurine, vinblastine, and systemic prednisone for 1 year, while mild SS-LCH has been treated with topical treatments such as nitrogen mustard or corticosteroids, or oral methotrexate. [4]. Due to its rarity, there has been no standard treatment plan for LCS, but surgical resection is an optimal option for localized disease, while etoposide, cyclophosphamide, doxorubicin, vincristine, and dexamethasone (E-CHOP) multiregimen has been used for systemic disease [26]. Histologically, LCH cells are 12–15 μm in diameter and have eosinophilic cytoplasm, irregular nuclei with prominent grooves and folds, and indistinct nucleoli [3].

Adult cutaneous LCH lesions are often located on the scalp, face, and external genitalia, and most commonly affect the flexural and intertriginous area, where they can manifest as pruritic skin lesions very similar to seborrheic dermatitis. Cutaneous lesions can also appear as red or purple nodules, erythematous papules, ulcerations, and abscesses. Severe acute forms can present as necrotizing plaques with pruritic and erosive lesions [2]. Cutaneous LCH has also been previously associated with diabetes insipidus (DI), a disorder of water balance in the kidneys characterized by polyuria and failure to concentrate urine. The etiology of DI comprises different autoimmune diseases, such as sarcoidosis, Wegner’s disease, infections, primary brain tumors, and lymphoma. Among diseases, LCH is the most common cause of DI—ranging between 15% and 29.1% [21, 30].

Given the rarity of LCH and its heterogeneous presentation, information about perianal cutaneous manifestations has been limited to case reports. Reported cases describe perianal lesions and systematic manifestations in the skeletal and pulmonary systems with their respective response to chemotherapeutic agents, with most patients being under the age of 50 years [2, 5–18]. Nevertheless, minimal information exists about natural LCH progression without treatment and disease management when an initial biopsy cannot exclude LCS in cases where patients are either not surgical candidates or not able to obtain surgical follow-up, limiting the ability to confirm underlying pathology. Here we describe the case of a 50-year-old Hispanic male with history of polyuria due to DI, chronic pruritus ani, and perianal ulcerations on examination who was ultimately diagnosed with LCH. We also review and compare his disease process with existing literature.

## Case presentation

A 50-year-old Hispanic male was referred to the colorectal clinic with a 9-month history of perianal itching and pain. He reported developing a rash as a result of severe itching. He was previously diagnosed with diabetes insipidus (DI) of unknown etiology and complained of frequent urination. He did not have any complaints of diarrhea, constipation, melena, hematochezia, changes in appetite, or weight loss. He had no family history of cancer. The patient had brain magnetic resonance imaging (MRI) in November 2017 that showed the absence of a hyperintense T1W signal of the neurohypophysis. He also had a colonoscopy in December 2019, which revealed a polyp in the transverse colon and perianal inflammation, which prompted his referral to the colorectal clinic. The polyp’s biopsy confirmed a benign adenomatous polyp. Surgical and medical history was otherwise negative.

On examination in the office, he was found to have a small amount of thick mucoid fluid around the anus, and significant inflammation of the perianal skin with radial excoriations involving the anal verge and extending 4 cm in radius. He also had nonblanching induration of the skin of his right leg below the knee and desquamation of his right hand.

**Fig. 1:**
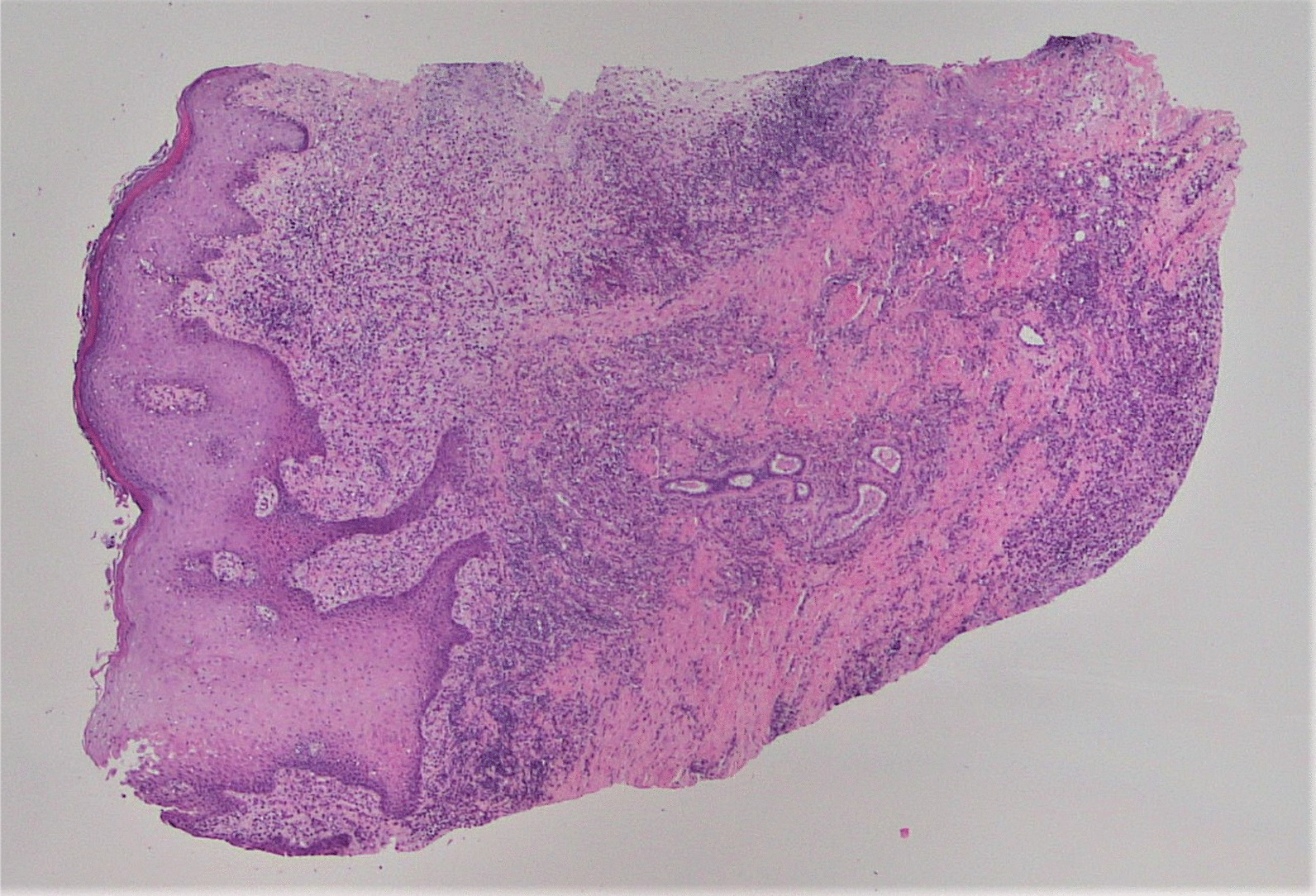
Hematoxylin and eosin (H&E) stain of perianal rash punch biopsy, 20× magnification

**Fig. 2:**
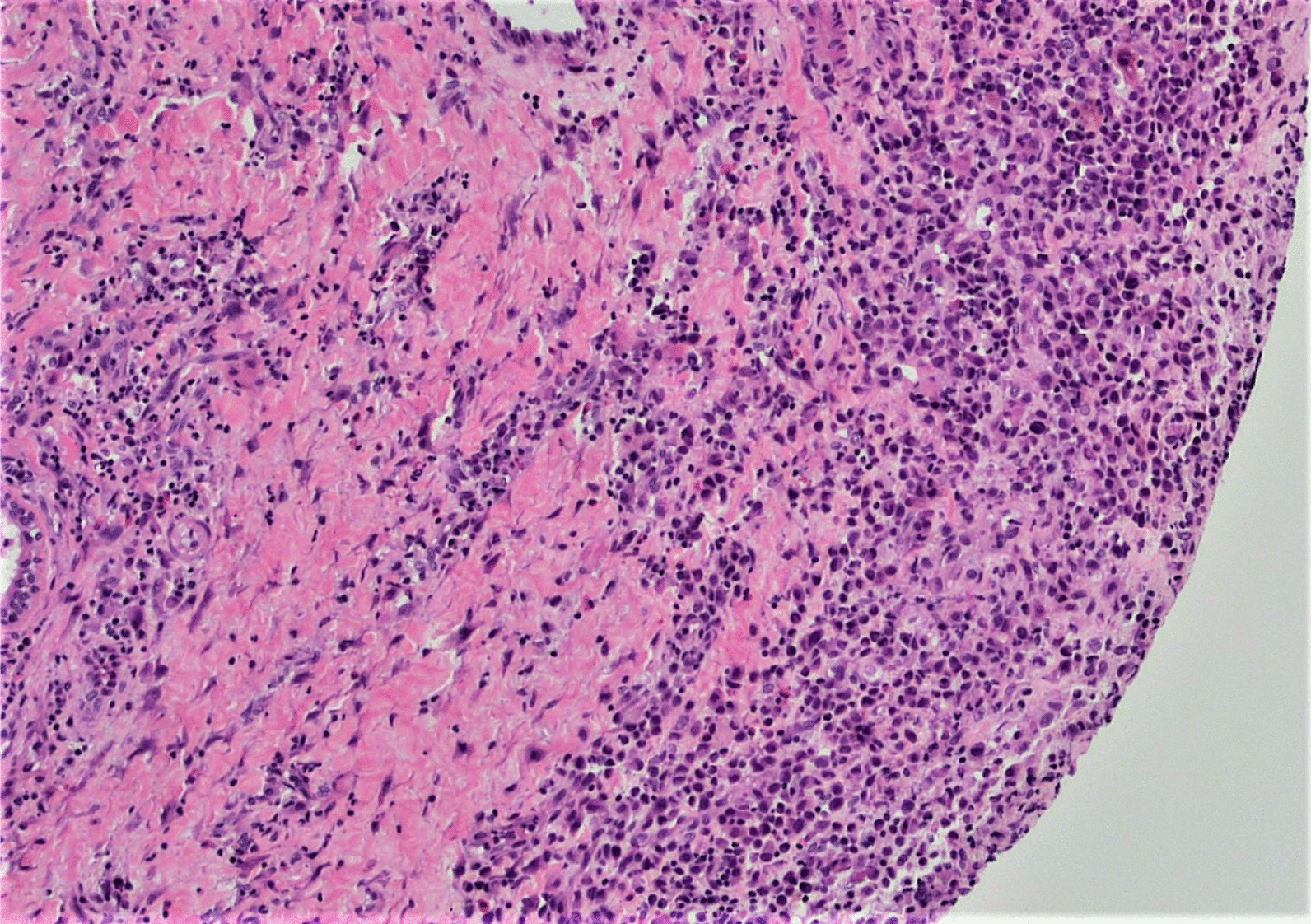
H&E stain of perianal rash punch biopsy, 100× magnification

**Fig. 3:**
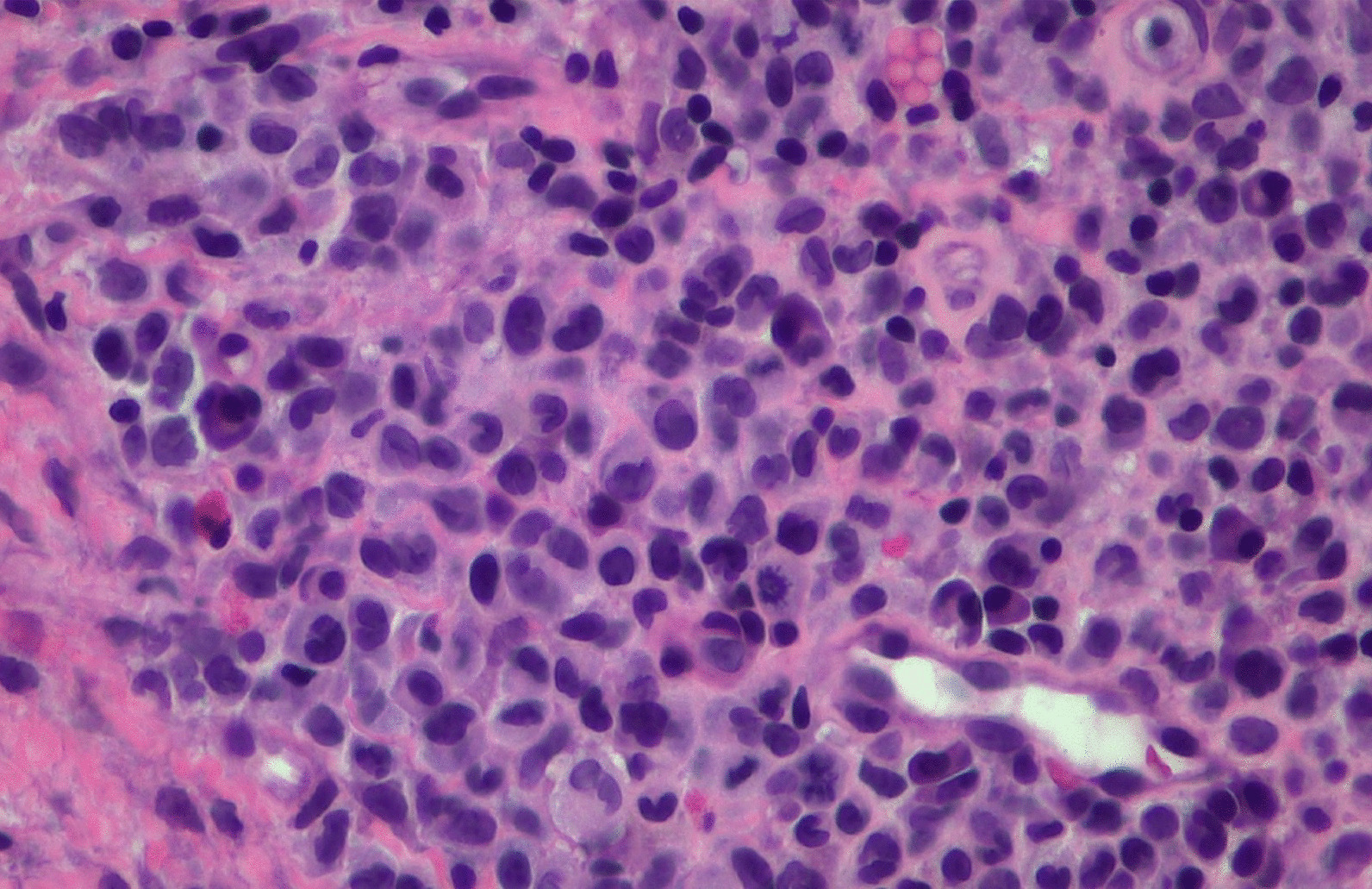
H&E stain of perianal rash punch biopsy, 400× magnification. Note the larger atypical plasmacytoid cells with mild-to-moderate nuclear pleomorphism, grooving, eosinophilic cytoplasm, and occasional mitotic figure

**Fig. 4:**
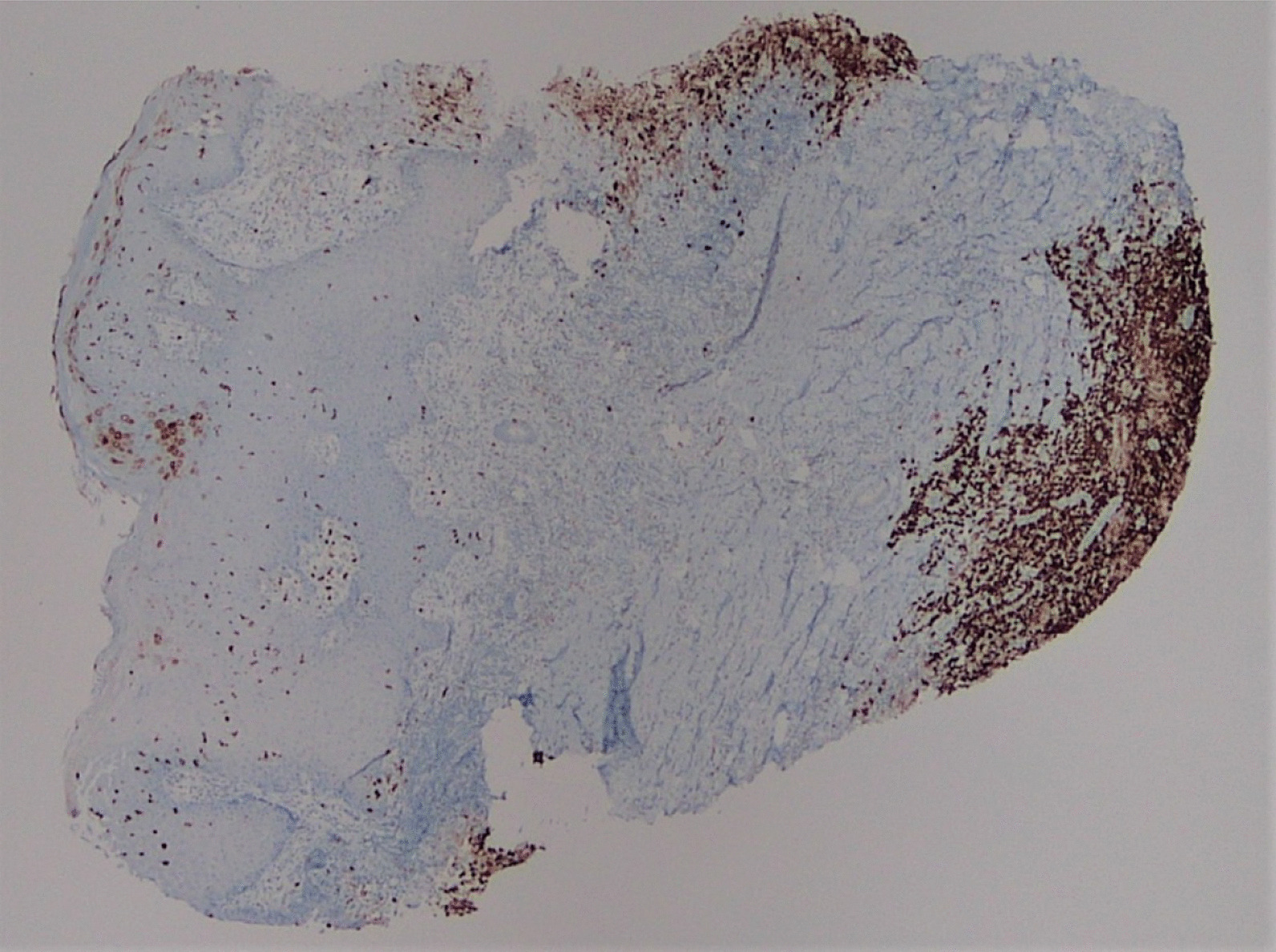
CD1a immunohistochemical stain, 20×, showing positivity in the Langerhans cells, extending to the base of the punch biopsy

**Fig. 5:**
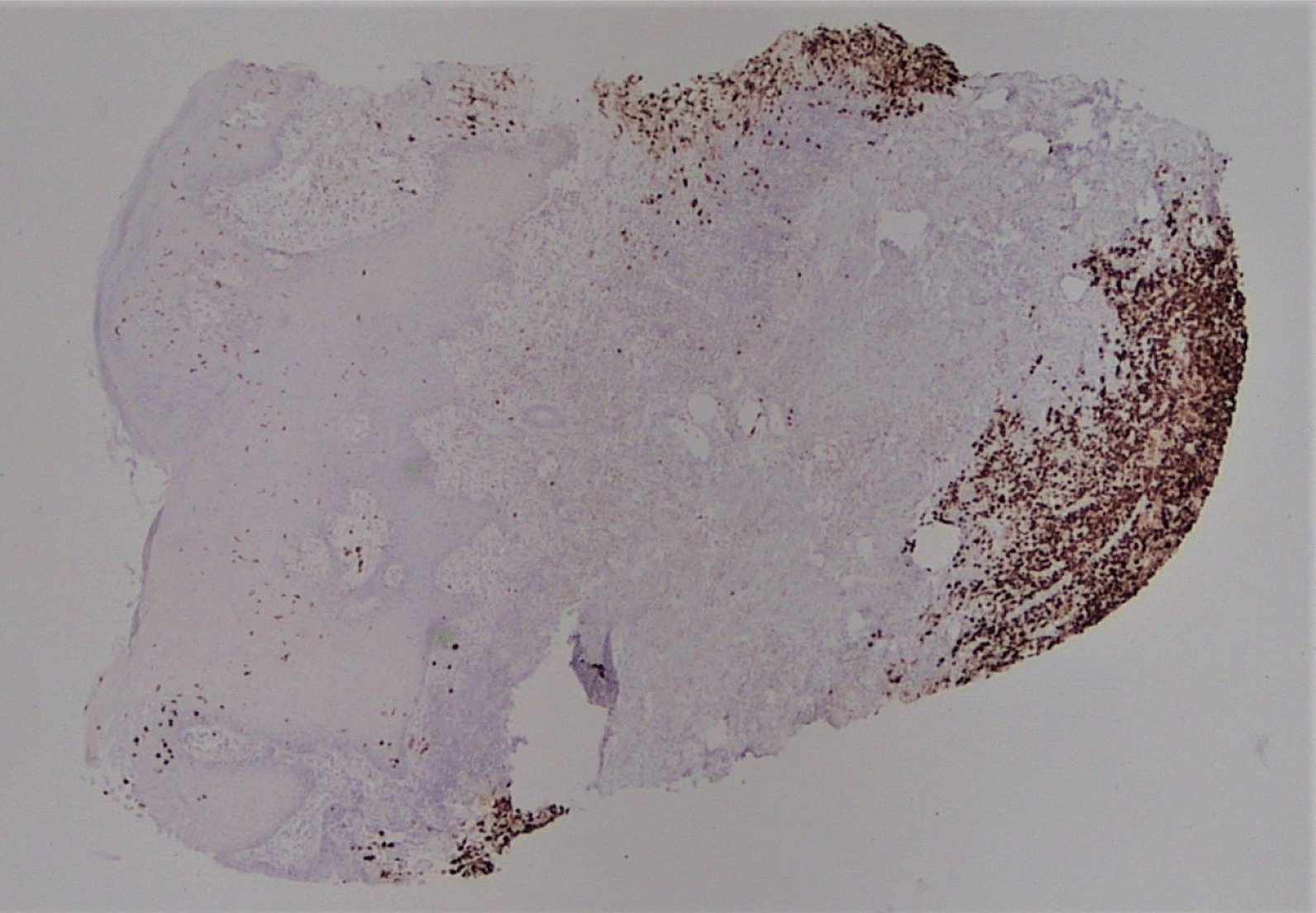
Langerin immunohistochemical stain, 20×

**Fig. 6:**
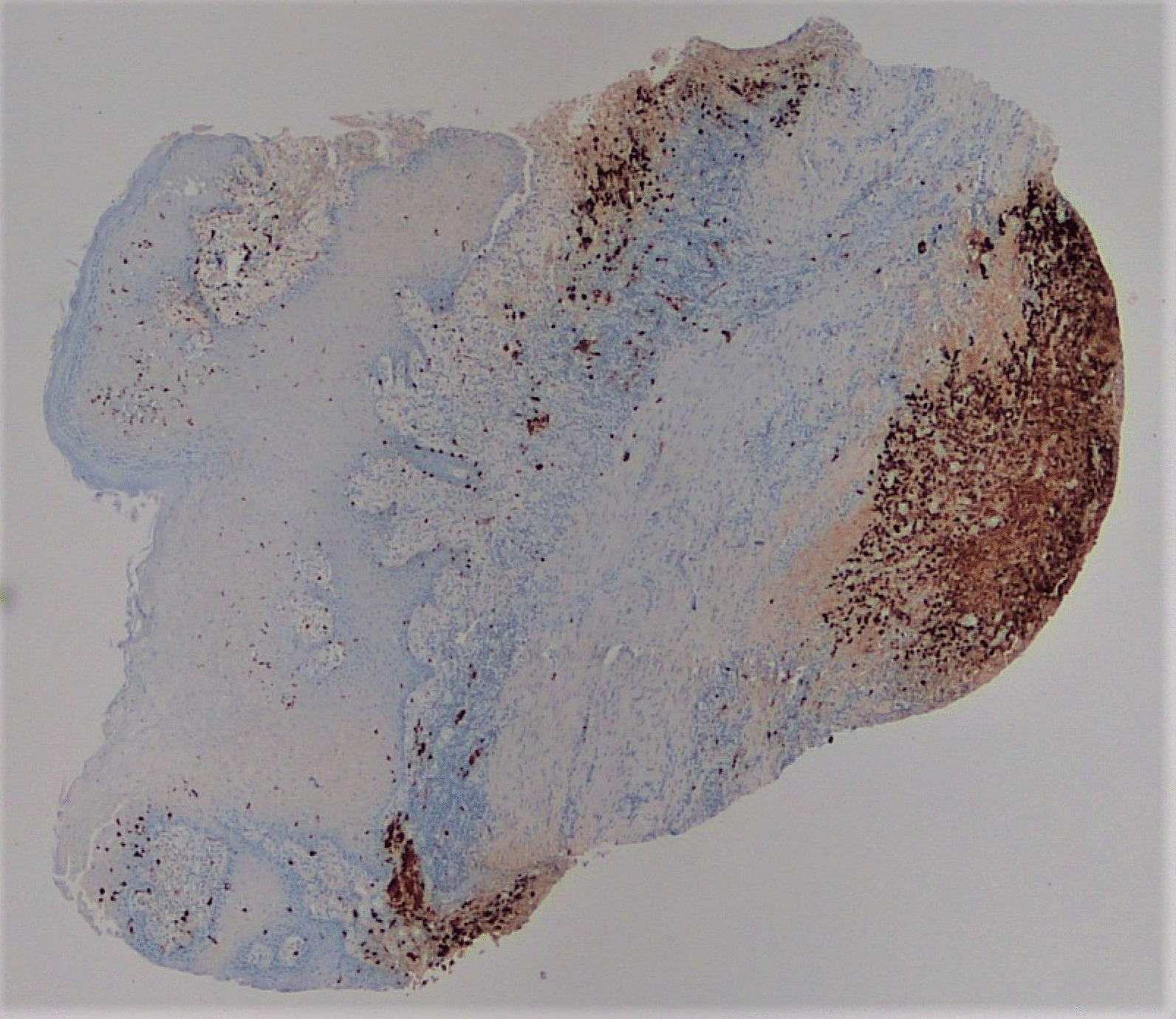
S-100 immunohistochemical stain, 20×

**Fig. 7:**
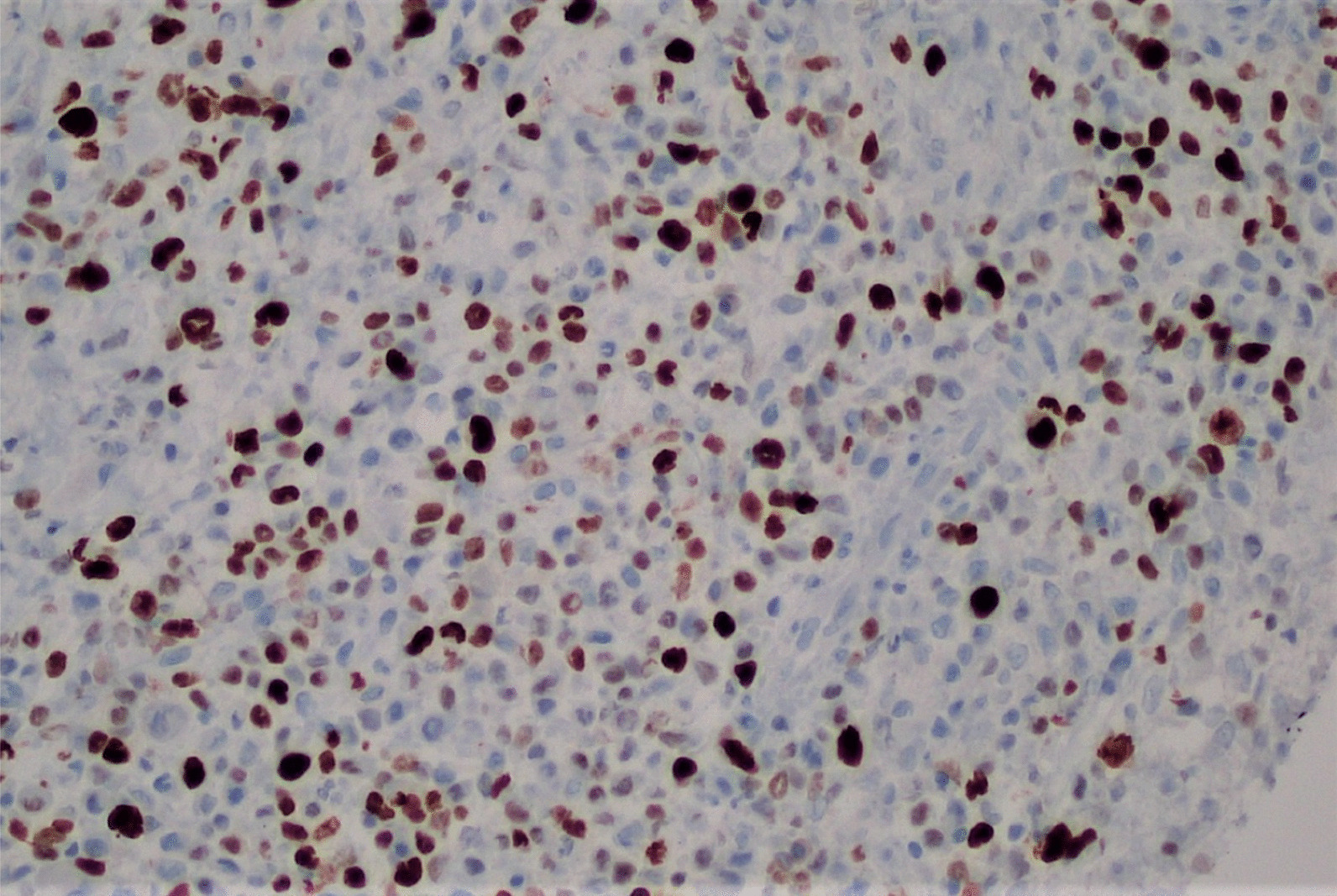
ki-67 index, showing a proliferation index of 50%

A punch biopsy was obtained at the intersection between the normal skin and the perianal rash during this initial visit. He was prescribed 1% zinc oxide cream to apply to the perianal skin for symptomatic management of pruritus three times daily. Pathology showed atypical plasmacytoid appearing cells in the deep dermis layer with mild-to-moderate nuclear atypia, mixed with a benign-appearing lymphoplasmacytic population with occasional eosinophils. The atypical cells were positive for S-100, CD1a, CD68, and Langerin, with a Ki-67 index of 50% (Figs. 1, 2, 3, 4, 5, 6, 7). These cells were negative for BRAF V600E mutations, CD3, CD20, carcinoma, and melanoma markers. Based on these results, he was diagnosed with a Langerhans cell neoplasm.

**Fig. 8:**
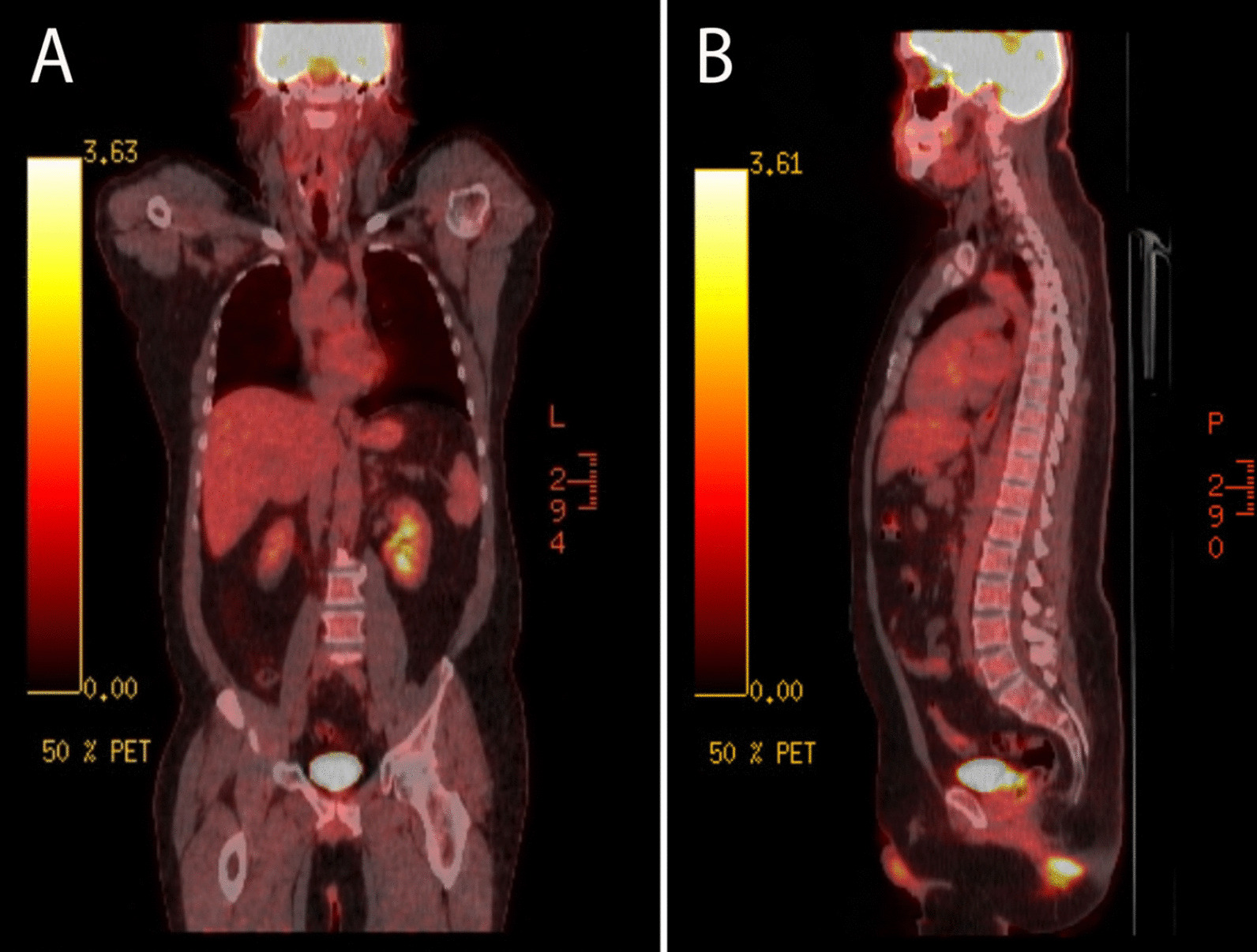
F-18 fluorodeoxyglucose (FDG)/PET scan in coronal (**A**) and sagittal views (**B**) showing physiological uptake

Three weeks later, the patient reported reduced pain and itching in his right lower extremity (RLE) and perianal area after using the topical zinc oxide cream. A positron emission tomography (PET) scan (Fig. 8) was also ordered to evaluate for any metastatic disease and failed to show any disease spread. Given the extent of disease that involved the anal margin, the patient would not have been a candidate for local excision as this would cause an anal stricture. The only surgical option would have been an abdominoperineal resection, committing the patient to a permanent end colostomy.

Given the localized nature of the disease, the patient was evaluated by an oncologist, who recommended local therapy with imiquimod. Of note, the patient lives in a medically underserved area, which presented specific challenges that have altered the patient’s care. This included delays in seeking and obtaining care as well as having limited resources available to facilitate surgical and medical care. Unfortunately, despite many attempts to contact the patient and prescribe this treatment, he was not able to follow up.

## Discussion

Langerhans antigen-presenting cells (LCs) belong to the histiocytic system and give rise to LCH and LCS. Due to their LCs’ origin, both LCH and LCS express CD1a, S100, and Langerin (CD207) proteins and show Birbeck granules on electron microscopy. LCH is benign and has a 5-year survival probability of 100% and 92% for single and multisystem disease, respectively [19, 20]. Initial treatments include topical steroids and local radiotherapy followed by systematic chemotherapy when refractory to initial measures [21]. Conversely, LCS is highly malignant and can form *de novo* or develop from an existing LCH. It is similar to LCH but is defined by its malignant cytological features of atypia, prominent nucleoli, and frequent mitotic figures [22]. It can disseminate into bones, lungs, spleen, gallbladder, and peritoneum; however, it can also present as a solitary skin lesion with or without lymph node involvement [23–26]. It has a bleak prognosis with a 2-year mortality of 52% and a median overall survival of 19 months [24, 27]. Surgical excision and chemotherapy are first-line treatment options for LCS [26, 28].

As the prognosis and respective treatments of LCH and LCS differ, it is important to obtain a sufficient biopsy sample to make a definitive diagnosis. Few studies have detailed specific parameters that differentiate LCS and LCH. Pileri *et al.*’s study of histiocytic tumors showed that LCH had mild-to-moderate nuclear atypia with benign-appearing nuclei, while LCS had frank malignant cytologic features with more prominent nucleoli [29]. Ki-67, a cellular proliferation marker, of the patient was elevated (50%), but he had mild atypia with no significant pleomorphism. This cellular morphology is consistent with a benign proliferation, but as biopsy was of limited size from the edge of the lesion, it is not possible to know the extent of cytologic malignant features, if present, and definitively exclude the possibility of LCS.

We depended on the clinical picture to guide the patient’s disease management. The patient’s perianal lesion has been stable since its eruption in June 2019, with no systematic metastasis despite the lack of chemotherapy or surgical intervention. In addition, the patient has a 3-year history of DI, which has been reported as an inaugural sign of LCH [30]. Between 10% and 16 % of LCH patients have DI [30, 31]. This is due to a particular predilection of LCH to the hypothalamic–pituitary axis (HPA), where monoclonal proliferation of aberrant histiocytes accumulates in and infiltrates into the axis. MRI findings associated with DI have been described. Typically, the pituitary stalk thickens, and it can progress into a mass lesion that extends to the hypothalamus and pituitary gland, and the normal hyperintensity of the pituitary is therefore lacking [32]. In Prosch *et al.*’s retrospective study, DI onset preceded LCH diagnosis in 43% of patients, and in 51% of these patients, LCH could be diagnosed within 1 year of DI [30]. There was no reported association between DI and LCS in the literature [22–24, 27, 31]. Thus, the patient was diagnosed with LCH based on his stable cutaneous lesion, history of DI, and benign cellular morphology.

We reviewed the literature and identified 15 adult patients with perianal LCH (Table 1) [2, 5–18]. The mean age was 38 years (range 18–70 years), with male predominance. LCH lesions were present from 4 months to 5 years before diagnosis, indicating a significant delay in identification and biopsy. In concordance with this case, 5/15 patients (33%) had a history of DI before LCH diagnosis, and 1/15 had it after. Patients were initially treated with antibiotics and corticosteroids, and systemic chemotherapy was started if the lesions were refractory. Three patients had surgical interventions: perianal lesion excision, diverting colostomy due to anal obliteration after a 10-year history of LCH ulcerations, and an abdominal perineal resection (APR) due to refractory perianal lesions [10, 11, 13]. Interestingly, six (40%) patients were in their thirties, two (13%) in their teens, two (13%) in their twenties, two (13%) in their forties, one (6%) in his sixties, and one (6%) in his seventies. While ample information describes LCH age distribution in children, limited information exists for adults. This provides new information regarding the age of perianal LCH presentation in adult patients.

**Table 1:** Cases of perianal Langerhans cell histiocytosis

Author, year	Age in years, sex	History	Perianal lesion morphology	Perianal lesion symptoms	Perianal lesion treatment	Interval^a^	Systemic involvement	Systemic treatment	Treatment outcome	Survival (follow-up time in months)
Abdou *et al.* 2017 [2]	33 M	Polydipsia (4 years), perianal lesions (2 years)	Perianal ulcerative plaque with raised edges oozing pus	Pain	Antibiotics and steroids	–	DI (no brain imaging obtained)	Methotrexate	–	–
Mansour *et al.* 2017 [5]	32 M	Polydipsia (10 years), Smoking	Cutaneous infiltration of anal sphincter, eroded ulcerative plaques over the anal orifice	Pain, bleeding and purulent discharge	None, only systemic	0	Lungs fibrosis; rectal tumor on colonoscopy, DI (no brain imaging obtained)	Vinblastine, prednisone, gemcitabine	Anal and colonic lesions, disappeared, lung fibrosis remained stable	Yes (6)
Gul *et al.* 2017 [6]	36 M	Anal fissure, two rectal surgeries	_	Pain and pressure	Local radiotherapy	9 months after perianal lesions	Thyroid	Vinblastine, prednisone, total thyroidectomy	–	Yes (6)
Bank *et al.* 1988 [7]	18 M	Itchy scalp lesion (2 years)	Ulceration with purulent secretions	–	Antibiotics	2 months after perianal lesions	Lung fibrosis and emphysema, bilateral (BL) pneumothorax, DI (no brain imaging obtained)	Vinblastine, prednisone, topical mustargen, lung subsegmental resection	Persistent scalp and perianal lesions	Yes (8)
Dere *et al.* 2016 [8]	45 F	Perianal wound (1 year)	3-cm ulcerovegetant purulent mass	Pressure sensation	Topical steroids	0	Femur and tibial lesions	Methotrexate (MTX)	Skin lesion healed with MTX	Yes (1 M)
Chauffaille *et al.* 1998 [9]	31 F	Polydipsia (3 years), vulvar ulcers (1 year)	Granulomatous perianal and vulvar ulceration	–	–	0	Femurs, skull, shoulder, forearm lesions, liver, oral lesions, DI, hypothalamic tumor on brain MRI	–	–	–
Mittal *et al.* 2009 [10]	45 M	Perianal ulcerations (4 M)	–	Painful perianal ulcerations	Nitrogen mustard, steroids, pentostatin. Then, surgical excision with APR and proctectomy	Prior to perianal lesions	Bone and lung	–	No recurrence of skin lesion post excision	Yes (36)
Foster *et al.* 2003 [11]	19 M	Perianal lesions (2 years), NSGY procedure	Two flat sessile perianal lesions on each buttock	Surface bleeding	Surgical excision	11 years prior perianal lesions	Extradural cranial mass invading cranial fossa on head computed tomography (CT)	Craniotomy for mass excision. Prednisone, vincristine, mercaptopurine	No recurrence of skin lesions and cranial mass	Yes (48)
Shahidi *et al.* 2011 [12]	20 M	Perianal wounds (1 year)	Well demarcate erythematous plaque	Difficulty in defecation	Antibiotics and steroids	None	None	Thalidomide	Lesion shrunk and became painless	Yes (6)
Waters *et al.* 2015 [13]	32 M	Perianal ulcerations (10 years), multiple excisions, and biopsies	Obliteration of anal verge by scar tissue	Pain and pressure	Laparoscopic diverting colostomy	None	None	None	–	–
Madnani *et al.* 2011 [14]	38 F	Polydipsia (8 years), vulvar ulcers (5 years)	Demarcated, indurated ulcer labia minora extending to the perianal area	–	Antibiotics multiple biopsies	0	Liver, bone, DI, mass in brain ventricle on brain MRI	Etoposide, 6-mercaptopurine, prednisolone	Bone pain disappeared, and skin lesions healed	Yes (120)
Conias *et al.* 1998 [15]	24 M	Scalp scaling and external auditory canal discharge and crusting (4 years)	Ulcerated hemorrhagic plaque extending to the scrotum (pain and bleeding)	–	Potassium permanganate, topical steroids, local radiotherapy	None	None	Cladribine	Scalp and ear lesions disappeared. Perianal lesions recurred and responded to local radiotherapy	Yes (12)
Field *et al.* 2007 [13]	70 M	Perianal pain and discharge (4 M), prostate CA	2-cm ulcerated lesion	Rectal bleeding, pain, mucous discharge	Steroids, potassium permanganate	After the perianal lesions	Tibial lesion	–	–	–
Broekaert *et al.* 2007 [17]	57 F	Polydipsia (2 years), inguinal and perianal ulcers (1 year)	Ulcerations with red raised border	–	None, only systemic	0	DI, thickened infundibulum on brain MRI	Thalidomide	Skin lesions healed; cerebral lesion remained stable	Yes (24)
Roeb *et al.* 2012 [18]	69 M	Perianal fistula, eczema, abdominal pain (4 years)	–	Perianal pain	Steroids	0	Colon (mucosal lesions throughout)	–	Perianal eczema and abdominal pain improved	–
This patient presentation	50 M	Polydipsia (3 years), perianal ulcers and pruritis ani (1 year)	Excoriated and erythematous, multiple superficial lacerations	Pruritus and pain	Silver sulfadiazine, zinc oxide	None	DI (no masses on brain MRI)	None	None	Yes

–, data not reported; Interval^a^, interval time between LCH lesions pathological diagnosis and systematic manifestation; *APR*, abdominal perineal resection; *DI*, diabetes insipidus; *MTX*, Methotrexate; *NSGY*, Neurosurgery; *CT*, Computed Tomography

## Conclusion

This is a unique case of LCH, wherein the patient presented with perianal pruritus ani that was diagnosed with LCH based on benign histological findings and the overall clinical course. All reported patients with perianal LCH have either underwent surgical intervention, were treated with systemic chemotherapy, and remained stable, or had LCH metastasis to bones, lungs, thyroid, and colon (Table 1), while this patient only had DI alongside the perianal rash. Stable perianal LCH lesion with benign clinical features in a 50-year-old male despite lack of additional medical and surgical treatment has not been described in the literature so far. This case provides information about the natural progression and variability of clinical course and outcome in patients with perianal LCH, and the review highlights patients’ age distribution, length of symptoms before diagnosis, modes of treatments, and respective outcomes in patients with perianal LCH.

## Limitations

This case report and patient is limited by the lack of surgical and oncological follow-up, which is far too prevalent in patients living in underserved communities. Fortunately, this patient’s disease remained stable, but the lack of appropriate care can lead to patients presenting with advanced disease that is seldom seen, otherwise. Ideally, the patient would have been seen in the colorectal clinic within a month of his colonoscopy and perianal inflammation findings that prompted his referral. He would have also been able to obtain a repeat biopsy, and then receive imiquimod treatment with close surgical and oncologic follow-up.

Learning points:

- A history of diabetes insipidus in a patient with a rash should raise a high index of suspicion, and the rash should be biopsied.
- LCH can disseminate into internal organs with a dismal prognosis, or it can be limited to a cutaneous lesion with a benign clinical course. Close follow-up is warranted to monitor progression.
- Caring for patients living in an underserved community adds a layer of complexity. Efforts should be done to track patients and facilitate appropriate follow-up.

## Data Availability

All data generated or analyzed during this study are included in this published article.

## Abbreviations

LCH: Langerhans cell histiocytosis
DI: Diabetes insipidus
MRI: Magnetic resonance imaging
PET: Positron emission tomography
LC: Langerhans cell
LCS: Langerhans cell sarcoma
SS-LCH: Single-system Langerhans cell histiocytosis
MS-LCH: Multisystem Langerhans cell histiocytosis
